# Knowledgeability about organic food consumption and the factors behind it

**DOI:** 10.3389/fnut.2023.1125323

**Published:** 2023-04-06

**Authors:** Sukanya Barua, Satyapriya, Rajeev Kumar, V. Sangeetha, L. Muralikrishan, Monika Wason

**Affiliations:** ^1^Division of Agricultural Extension, ICAR-Indian Agricultural Research Institute, New Delhi, India; ^2^Division of Agricultural Engineering, ICAR-Indian Agricultural Research Institute, New Delhi, India

**Keywords:** organic food, knowledge level test, Delhiites, consumers, farmers, health consciousness

## Abstract

**Introduction:**

The popularity of organic food is in increasing trend due to the increased existence of synthetic food worldwide. Hence it is very important to know the knowledge level of city dwellers regarding various aspects of organic food and factors associated with their awareness level.

**Objectives:**

The primary objective of this research study is to develop a standardized knowledge test to assess the knowledge level of respondents regarding organic food and find out the factors behind elevated knowledge levels.

**Methods:**

A standardized knowledge test was developed comprising 26 knowledge items and pilot tested on 42 individuals. Difficulty index, discrimination index and point biserial correlation coefficient were calculated; only 21 knowledge statements were selected out of 26. The reliability coefficient and validity were checked and found satisfactory. The final knowledge test containing 21 knowledge statements was administered to 1050 respondents from various locations of the National Capital Region (NCR)-Delhi, India. After getting the knowledge score from each individual, it was classified as very low, low, medium, high and very high knowledge level. For determining factors contributing towards enhanced knowledge level, the correlation coefficient was calculated between independent socio-economic variables of each individual and their corresponding knowledge score. Regression analysis was also performed and developed a model to depict a relationship between the dependent variable i.e. knowledge level and independent variables.

**Results:**

Standardized knowledge test depicted that a major portion of respondents (62.0%) possessed very low, low and medium levels of knowledge, whereas 23.5 and 14.5% of respondents had high and very high levels of knowledge regarding organic food. Independent variables like gender, education, family size, family income, internet, mass media exposure and social participation had a positive relationship with the knowledge level of respondents. The results of regression analysis show that education (X_2_), total annual income (X_3_), gender (X_6_), participation in various organizations like the club, societies, etc. (X_8_), health consciousness of individual (X_11_), perception of organic food (X_13_); could explain the major share of ~62.1% of the variation in dependent variable i.e. knowledge level.

**Conclusion:**

The developed standardized knowledge test for the present study was found valid and appropriate research tool for evaluating the knowledge level of urban citizens regarding organic food. The majority of respondents had a positive attitude towards organic food but possessed low to medium knowledge levels regarding organic food. Occasional awareness campaigns and capacity-building programs regarding various aspects of organic food in educational institutes, residential societies and through mass media can be beneficial to society.

## Introduction

The consumption of organic food has increased among consumers all over the world since last decade. Our country has a 5,28,261 ha area under organic farming, which includes 44,921 no. of certified organic farms (1). This accounts for ~0.3% of total agricultural land. The agricultural and Processed Food Products Export Development Authority (APEDA) reported that approx. 5,85,970 tons of organic products worth INR 301 million are exported from India (2). Increasing awareness, growing market demand, the high interest of farmers to grow organic food and increasing institutional support have resulted in tremendous progress in certified land (3, 4). The high purchasing pattern is determined by an increased level of consumer awareness of safe food and health concerns (5, 6). The demand for nutritious organic food is based on its quality (7, 8). While the demand for organic food is increasing, the popularity of organic food is not widespread and consumers’ perception of organic food is varied (9). There is variation in people’s understandings of organic agriculture practices and consumers’ attitudes, motivations and behaviors toward organic food (10, 11). Global markets are becoming increasingly concerned with chemical-free food production systems driven by health, nutritional and environmental concerns (12, 13). Hence, it is necessary to know about the knowledge level of consumers of metropolitan cities like Delhi, mainly urban consumers demand organic food. The behavioral dimension of the farming community is related to solely their interest, economic benefit and other livelihood-related issues (14). Sustainability, competitiveness and favorable government policy can be key roles behind a successful organic food market (6).

Developed countries like Japan, European countries, Germany and the United States reported that recent consumer trends inclined toward less intensively made food (15). Diminishing public interest in the modern method of farming and post-harvest technologies and growing public knowledge on food-related hazards such as antibiotics, pesticides, hormones, and artificial ingredients is the main reason (16, 17). This increasing demand in the global market can be met with increased organic farms. As consumers’ purchasing trend is governed by knowledge and awareness level, it is imperative to assess them. Hence, a study was conducted in NCR–Delhi (India) to ascertain the level of knowledge possessed by Delhiite respondents about important factors determining it.

## Materials and methods

### Development of knowledge test and details of the study area

**Figure 1:**
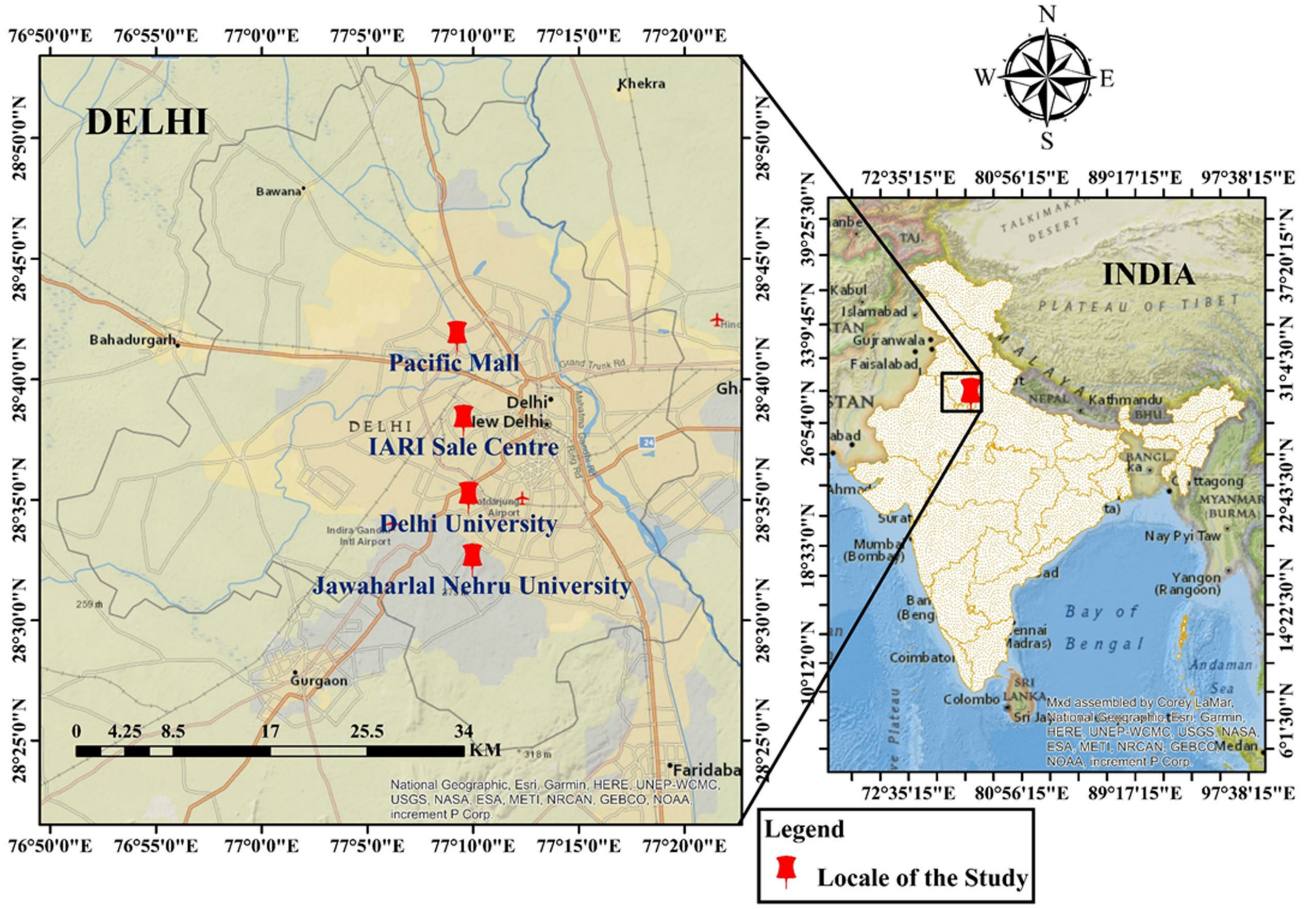
Study area of Delhi showing selected markets/consumer zones for the knowledge test.

The study was conducted during 2016–2019 under the IARI Institutional Project and UNDP-funded project entitled “Strengthening Agri-Nutri linkage for enhancing nutritional security and empowering farm women in India: Leveraging Agriculture for Nutrition”. A knowledge test was constructed to measure the knowledge level of organic food among urban consumers and a pilot test for this was done among 42 individual respondents. Twenty-six knowledge items that reflect various aspects of organic food were enlisted for the execution of the test. Each test item was considered based on relevant organic food concepts from relevant literature and expert suggestions. Selected 26 knowledge items were given to the concerned experts of the ICAR–IARI, New Delhi, India and were mailed to other experts outside Institutes too to check their appropriateness. Experts were asked to give remarks and modify statements/items if required. Respondents came from different parts of Delhi as the study area (Figure 1) in randomly selected markets/consumer zones *viz.* Pacific mall (Public) (Latitude & Longitude ~28.6948, 77.1542), IARI sale center (scientists and outsiders) (28.6374, 77.1593), Delhi University-New Delhi (Staff and research scholars) (28.5843, 77.1638), and Jawaharlal Nehru University-New Delhi (Staff and research scholars) (28.5398, 77.1664) were selected for the pilot and final knowledge test (Figure 1).

The preliminary test consisting of 26 knowledge items (Refer to Table 1) was administered to 42 respondents representing various parts of Delhi and these items were analyzed using statistical indexes. Score ‘1’ was assigned to the right answer and ‘0’ to the wrong answer. The obtainable score varied from 0–21 for each respondent. Three indexes, i.e., difficulty index, point biserial correlation coefficient, and discrimination index, were calculated for the final selection of items.

**Table 1:** Item analysis of questionnaire for knowledge testing about organic food among Delhi respondents (*n* = 42).

S. No.	Knowledge items	Difficulty index	Discrimination index	Point-biserial correlation coefficient
1	Familiarity of “organic agriculture” or “organic food”	52.38	0.50	0.520
2	Practical experience with organic farm	47.62	0.21	0.475
3	Criteria regarding organic food and farming	45.24	0.24	0.447
4	Mobile application regarding organic farming	54.76	0.24	0.545
5	Safety of organic food	50.79	0.93	0.500
6	Identification of organic food	52.98	0.36	0.523
7	Heavy metal concentration limit in organic food	49.21	0.86	0.490
8	Indian organic certification mark	52.38	0.64	0.518
9	Criteria for organic livestock population	53.17	0.43	0.430
10	GMO and organic food	53.57	0.36	0.520
11	Nutrients for organic foods	53.17	0.79	0.430
12	Organic foods and conventional foods	49.17	0.40	0.482
13	Nutritive value of organic food as compared to traditional food	38.97	0.39	0.379
14	Price of organic food as compared to conventional food	41.38	0.42	0.489
15	Factors behind organic food is safer	35.27	0.59	0.451
16	Identification of ‘logo’ in organic food	71.35	0.34	0.457
17	The place to buy organic food in Delhi	58.29	0.44	0.399
18	Best practices of organic food	40.53	0.25	0.478
19	The complexity of practices in growing organic food	45.71	0.31	0.396
20	Environment safety of organic foods	57.82	0.38	0.478
21	Criteria for defining organic food	67.21	0.41	0.529

### Difficulty index (*P*)

The difficulty index measures the degree of complexity of an item or indicates how much a question is difficult. A question (or item) should not be too simple that everyone can pass, nor too hard that no one can pass. In this study difficulty index is symbolized as *P*. The mentioned formula calculated the difficulty index (*P*):


$$P=\frac{\mathrm{NC}}{N}\times 100$$

NC = the number of samples who correctly answered, *N* = Total number of sample sizes. Items having a *p* value within 30–80 were selected for the final knowledge test (18, 19). *p* value was calculated for each statement/item separately.

### Discrimination index

The discrimination index was also calculated for item selection. This index determines whether an item/question has discriminating power to differentiate between well-informed and not-informed individuals.

Similar to the difficulty index, the discrimination index was also calculated for each statement. Scores obtained for each statement (by adding scores of subcategories of each statement) from all the samples were arranged in descending order and scores were divided into six groups (7 respondents in each group) i.e., G_1_, G_2_, G_3_, G_4_, G_5_, and G_6_. The middle two groups, i.e., G_3_ and G_4_ were deleted and the four terminal groups, i.e., high-score groups (G1 and G2) and low-score groups (G_5_ & G_6_) were kept for further analysis. DI was calculated for each statement separately by using the following formula:


$$\mathrm{DI}=\frac{\left(S1+S2\right)-\left(S5+S6\right)}{N/3}$$

Where, DI = Discrimination index, S_1_, S_2_, S_5_, & S_6_ are the frequencies of right answers in group of G_1_, G_2_, G_5_ and G_6_, correspondingly. *N* is the total number of samples for item analysis. The present study selected the statement with a discrimination index value of more than 0.20 (18) for the final knowledge test item.

### Point-biserial correlation coefficient (*r_pbi_*)

The point-biserial correlation coefficient (*r_pbi_*) was estimated to calculate the internal consistency of each statement. It was determined using the following equation (19, 20):


$$r_{pbi}=\frac{X_{p}-X_{q}}{S_{t}}\times \sqrt{\mathrm{pq}}$$

Where, *r_pbi_* = Point-biserial correlation coefficient, *X_p_* = Mean score of variable in the successful group, *X_q_* = Mean score of variable in the unsuccessful group, *S_t_* = Standard deviation of the total sample, *p* = Percentage of samples who answered correctly and *q* = percentage of respondents who answered incorrectly or not answered. The final knowledge test selected a point biserial coefficient equivalent or more than 0.235 (18).

Finally, 21 statements were selected out of 26 for final inclusion in the knowledge test.

### Reliability of test

To check the reliability of the test, the Split-Half method was performed on respondents. The pilot test sample was distributed into equal halves and a questionnaire was administered to respondents. The total score of each half of the respondents was correlated using the Spearman-Brown formula. The coefficient was found to be 0.85 which is considered highly significant. The reliability coefficient is determined by using the following equation (21):


$$r_{tt=\frac{2\;r_{\mathrm{hh}}}{1+r_{\mathrm{hh}}}}$$

Where, *r_tt_* = reliability coefficient and *r_hh_* = the correlation between two halves of the sample.

### Standardized knowledge index

The index was calculated by using the following formula:


Standardized Knowledge Index(KI)=Knowledge score obtainedbyrespondentMaximum obtainable knowledge score×100

The standardized knowledge index was calculated by assigning a score of “1” against the right answer and “0” against the wrong answer correspondingly. The maximum score of the respondent can score is “21”. Based on the score in the knowledge index, the respondents were divided into the following five groups of the level of knowledge, respectively, (19) i.e. Very low (<5.045), Low (5.045–10.090), Medium (10.091–15.135), High (15.136–20.18) and Very High (>20.180).

### Knowledge level of Delhiites about organic foods of 42 respondents

A standardized test was developed in the present study to measure respondents’ knowledge level about organic foods. A total of 21 items were categorized into 3 sub-heads of product knowledge *viz.* (i) Basic concepts of organic foods, (ii) identification criteria and properties of organic foods and (iii) differences between organic and inorganic foods. Three parameters of item analysis, i.e., difficulty Index, discrimination index and point-biserial correlation coefficient of 21 items of 42 respondents were determined which is tabulated in Table 1. The preliminary survey respondents were not included in the final test to evade the pre-testing effect.

These indexes were calculated for the selection of items in the knowledge test. From a total of 26 statements/items, 21 items were chosen based on a difficulty index ranging from 30 to 80, a discrimination index of more than 0.20 and a biserial coefficient equal to or more than 0.236 as shown in Table 1.

Multiple regression analysis was performed to depict the relationship between knowledge regarding organic food and other independent variables. The prime objective of multiple regressions is to use those independent variables whose values are known to predict the single dependent variable value. The Multicollinearity situation may exist when independent variables in the regression model are highly correlated to each other. It makes it hard to interpret of model and also creates an overfitting problem. So it is necessary to calculate the Collinearity value and Variance Inflation Factor (VIF). VIF is a measure of the amount of multicollinearity in regression analysis. Variables with a VIF of less than 5 will be included in the model. However, many sources say that a VIF of less than 10 is acceptable. The correlation coefficient (Pearson’s) was calculated to determine the relationship between the level of knowledge and their socio-personal variables at a 1% level of significance. The SPSS-16 software was used for the analysis of data.

## Results and discussion

**Figure 2:**
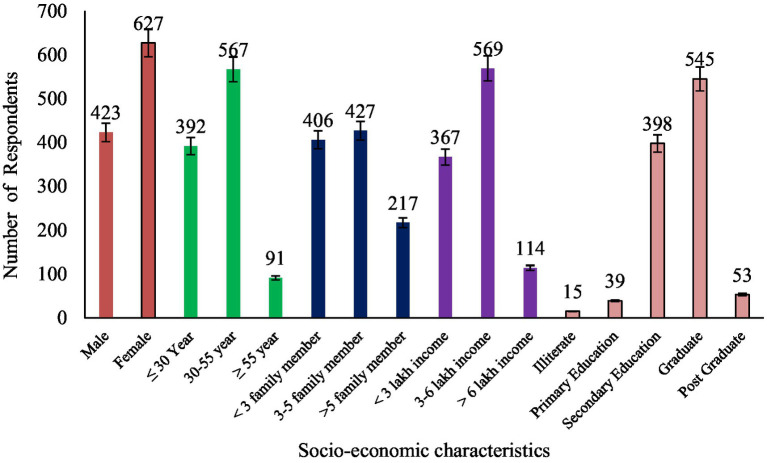
Socio-economic profile of respondents (*n* = 1,050).

A developed knowledge test of 21 items regarding organic food was executed in the same locale from different parts of Delhi, as shown in Figure 1, on 1,050 respondents (n = 1,050). The classification of these respondents based on gender, age, income, education and their number in each respondent group is shown in Figure 2. The knowledge index of 1,050 respondents was categorized as very low, low, medium, high, and very high which is depicted in Table 2. The table shows only 23.5 and 14.5% of Delhiite respondents had high and very high levels of knowledge about various aspects of organic food, while most of the respondents lying in the low to medium category, i.e., 45.5% followed by 16.5% in the very low-level knowledge category. Hence, we can conclude that more than half of the respondents have very low to medium knowledge levels and awareness. The mean of knowledge indexes about organic food was calculated as 51.1%, which is medium. Divakar et al. and Yadav et al. report similar type findings by using knowledge tests in both of their studies (18, 22).

**Table 2:** Distribution of level of knowledge about organic food by the Delhiites (*n* = 1,050).

Level of knowledge	Frequency (*f*)	Percentage (%)
Very low (<5.045)	174	16.5
Low (5.045–10.090)	263	25.0
Medium (10.091–15.135)	215	20.5
High (15.136–20.18)	246	23.5
Very high (>20.180)	152	14.5

Pearson’s correlation coefficient was calculated and presented in Table 3. The correlation study revealed that gender, education, family size, family income, internet, mass media exposure, and social participation had a positive relationship with the knowledge level of respondents. Kumar et al. and Shakya et al. also found a positive association between education and respondents’ knowledge level because educated consumers are more aware of safe food consumption (23, 24). Furthermore, family income was significantly associated with the respondents’ organic food knowledge because consumers having more income had the urge to change their food consumption pattern toward safe and natural food though it is costly. Smith et al. also found that income and organic food consumption behavior are positively correlated with each other (25). Likewise, these results also showed a positive and significant correlation between internet exposure, mass media exposure and social participation in various social organizations with organic food knowledge (11, 18). Various contacts and access to information make people more aware of safe food. The background of rural or urban people does not significantly influence organic food knowledge. Food habits (vegetarian/non-vegetarian) also do not significantly influence consumers’ knowledge about organic food. Health consciousness, consumers’ buying behavior and perception of organic food significantly correlated with knowledge level. Segovia-Siapco also concluded in a similar observation (26).

The results of regression analysis are presented in Table 4 shows the most important variables affecting the level of knowledge of Delhiites about organic food. The results indicated that six independent variables *viz.* Education (X_2_), Total annual income (X_3_), Gender (X_6_), participation in various organizations like the club, societies, etc. (X_8_), health consciousness of individual (X_11_), perception of organic food (X_13_); could explain the major share of ~62.1% of the variation.

**Table 3:** Association between the socio-personal variables of Delhiite respondents and their knowledge level (*n* = 1,050).

Variables	Correlation coefficient
Age	0.0540
Education	0.3429**
Gender	0.2501**
Family income	0.0435
Occupation	0.1094
Family size	0.2588**
Marital status	0.0443
Internet exposure	0.5952**
Social participation	0.3514**
Mass media exposure	0.0112
Background	0.3420**
Food habit	0.0518
Health consciousness	0.5723**
Buying behavior	0.3782**
Perception about organic food	0.2534**

** indicates the significant relationship.

**Table 4:** Regression co-efficient and collinearity statistics of knowledge level about organic food (*n* = 1,050).

Code	Characteristics	*R* ^2^	Regression coefficient	Standard error	*t*-value	Collinearity value	VIF
X_1_	Age	0.005	0.058	0.06	0.99	0.422	2.371
X_2_	Education	0.020	0.789**	0.39	2.03	0.368	2.715
X_3_	Total annual income	0.012	0.899**	0.26	2.55	0.590	1.694
X_4_	Family type	0.002	1.118	1.82	0.61	0.703	1.423
X_5_	Family size	0.050	1.135	1.61	0.71	0.546	1.831
X_6_	Gender	0.170	1.101**	0.27	4.12	0.380	2.634
X_7_	Mass media exposure	0.001	0.278	0.76	0.36	0.441	2.265
X_8_	Membership in social organization	0.241	1.397**	0.29	4.81	0.640	1.562
X_9_	Background	0.023	0.296	0.40	0.73	0.658	1.319
X_10_	Food habit	0.085	0.433	0.23	1.85	0.501	1.996
X_11_	Health consciousness	0.055	0.0001	0.44	1.42	0.550	1.819
X_12_	Buying behavior	0.115	0.628**	0.00	2.71	0.588	1.252
X_13_	Perception about organic food	0.241	1.397**	0.29	4.81	0.647	1.341

** indicates the variables significant at 1% level of confidence.

*Y* = 0.7889×_2_ + 0.8995×_3_ + 1.1006×_6_ + 1.397×_8_ + 0.0001×_12_ + 1.3967×_13_.

Table 4 also depicts the collinearity statistics which can be defined as several independent variables correlated among them. A variance Inflation Factor (VIF) is a measure of the amount of multicollinearity in regression analysis. A VIF value less than 5 (27) indicates a low correlation of that independent variable with other predictor variables. For all the independent variables in our study VIF value less than 5, indicates no significant multicollinearity situation. So no variable was dropped due to the multicollinearity problem. A collinearity value of more than 0.7 indicates the presence of multicollinearity and no variable presented here shows the Collinearity problem.

## Conclusion

It is concluded that the standardized knowledge test developed for the present study was found valid and appropriate research tool for evaluating the knowledge level of residents of NCR–Delhi region regarding organic food. It was also revealed that the majority of samples possessed low to medium knowledge levels regarding organic food. While most of the respondents have basic knowledge and a positive attitude toward organic food, the majority of them possess inadequate in-depth knowledge about various specifications of organic food and guidelines about organic farms. So, effort needs to educate people about organic food and how to identify the original one. Education, income, gender, social organization membership, buying behavior, and perception about organic food were found significantly influences the knowledge level of urban organic food consumers.

Research on organic food possesses a lot of scope in today’s era due to the rise in many complex diseases. Indiscriminate use of chemicals in food right from cultivation to post-harvest procedure paves the way for dangerous health complications. So knowledge regarding proper identification of organic food, distinct characteristics from non-organic ones and limitations of organic food for rural as well as urban consumers need to be assessed. Analysis of buying behaviors and readiness to pay can be a great researchable issue for city dwellers. Occasional awareness programs and capacity-building programs in educational institutes, residential societies and through mass media can be options to enhance communities’ awareness levels.

## Data availability statement

The raw data supporting the conclusions of this article will be made available by the authors, without undue reservation.

## Ethics statement

Ethical review and approval was not required for the study on human participants in accordance with the local legislation and institutional requirements. Written informed consent for participation was not required for this study in accordance with the national legislation and the institutional requirements.

## Author contributions

SB: conceptualized, designed and wrote the manuscript. SP: designed and collect the data. RK: collect the data, review of literature, referencing and made GIS graphics. VS: analyzed the data and edited the manuscript. LM and MW compiled and curation of collected data. All authors have read and agreed to the published version of the manuscript.

## Funding

This Research is to carry out under the IARI Institutional Project and UNDP funded project.

## Conflict of interest

The authors declare that the research was conducted in the absence of any commercial or financial relationships that could be construed as a potential conflict of interest.

## Publisher’s note

All claims expressed in this article are solely those of the authors and do not necessarily represent those of their affiliated organizations, or those of the publisher, the editors and the reviewers. Any product that may be evaluated in this article, or claim that may be made by its manufacturer, is not guaranteed or endorsed by the publisher.
